# Biomimetic and Bioinspired Materials: Design Strategies, Mechanical Properties, and Engineering Applications—A Review

**DOI:** 10.1002/gch2.70101

**Published:** 2026-03-27

**Authors:** Manickaraj Karuppusamy, Sivasubramanian Palanisamy, Mahesh Gurusamy, Vijaya Prakash Balasubramanian, Manivannan Jayamani, Subramanian Lakshmi Sankar, Karthikayan Sundararajan, Gokulkumar Sivanantham, Syed Kashif Ali, Murugesan Palaniappan, Mezigebu Belay, Mohamed Abbas

**Affiliations:** ^1^ Department of Mechanical Engineering CMS College of Engineering and Technology Coimbatore Tamil Nadu India; ^2^ Department of Mechanical Engineering School of Engineering Mohan Babu University Tirupati Andhra Pradesh India; ^3^ S.K.TECHNOLOGYS, Guru Industrial Park, Ranga Nagar D Block Coimbatore Tamil Nadu India; ^4^ Department of Mechanical Engineering Dhanalakshmi Srinivasan College of Engineering Coimbatore Tamil Nadu India; ^5^ Department of Mechanical Engineering Kalasalingam Academy of Research and Education Krishnankoil Tamil Nadu India; ^6^ Department of Mechanical Engineering Sathyabama Institute of Science and Technology Chennai Tamil Nadu India; ^7^ Department of Mechanical Engineering KPR Institute of Engineering and Technology Coimbatore Tamil Nadu India; ^8^ Department of Physical Sciences Chemistry Division College of Science Jazan University Jazan Kingdom of Saudi Arabia; ^9^ Engineering and Technology Research Center Jazan University Kingdom of Saudi Arabia; ^10^ Department of Mechanical Engineering College of Engineering Imam Mohammad Ibn Saud Islamic University (IMSIU) Riyadh Kingdom of Saudi Arabia; ^11^ Department of Metallurgical and Materials Engineering College of Engineering Ethiopian Defence University Bishoftu Ethiopia; ^12^ Electrical Engineering Department College of Engineering King Khalid University Abha Saudi Arabia; ^13^ Department of Condensed Matter Physics Saveetha School of Engineering Saveetha Institute of Medical and Technical Sciences (SIMATS) Chennai India

**Keywords:** bioinspired design, biomimetic materials, engineering applications, mechanical performance, synthesis techniques

## Abstract

The field of biomimetic and bioinspired materials has progressed rapidly by drawing inspiration from nature's intricate structures and multifunctional systems in order to address pressing challenges in modern engineering. This review critically examines the mechanical performance of these materials, focusing on their hierarchical design principles, synthesis strategies, and versatility of application. Natural exemplars such as nacre, spider silk, and bone are emphasized, as their structural efficiency and functional adaptability have informed the development of synthetic analogues with superior strength, toughness, flexibility, and lightweight characteristics. The review also highlights advanced fabrication methods, such as additive manufacturing and precision chemical synthesis, which have enabled researchers to replicate the complexity of nature with ever‐greater accuracy. Key case studies demonstrate how bioinspired strategies have been translated into high‐performance materials for use in the aerospace, construction, and biomedical sectors. The review also discusses challenges related to scalability, reproducibility, and industrial integration. Finally, the review outlines emerging interdisciplinary approaches that are set to further enhance the mechanical properties and practical relevance of biomimetic materials, establishing them as transformative solutions for the next generation of engineering systems.

## Graphical abstract

This graphical abstract presents biomimetic materials inspired by natural systems such as nacre, spider silk, lotus leaves, and bone. It highlights hierarchical structuring, self‐healing mechanisms, and functionally graded composites. Fabrication techniques include additive manufacturing, CVD, self‐assembly, and bioreactors. Applications span aerospace, biomedical engineering, construction, and textiles, emphasizing lightweight strength, durability, sustainability, and multifunctional performance.

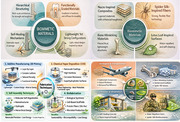

## 1 Introduction

The field of biomimetic and bioinspired materials has gained significant attention in recent years due to its ability to replicate nature's highly efficient structural and functional designs. Biomimicry, the practice of drawing inspiration from natural systems and biological organisms, has led to the creation of materials with exceptional mechanical properties, including high strength, toughness, flexibility, and lightness. [1]. Over millions of years of evolution, nature has optimized materials for survival, efficiency, and adaptability, resulting in structures that outperform many of the artificial materials engineered by humans. Natural materials exhibit unique hierarchical architectures that contribute to their mechanical superiority. Spanning multiple length scales from the molecular to the macroscopic, these architectures allow materials to exhibit extraordinary toughness, resilience, and functional versatility [2, 3]. By studying and emulating these biological structures, engineers and scientists have developed innovative materials that not only match but, in some cases, surpass the performance of conventional synthetic materials. Biomimetic materials can be tailored for specific applications, ranging from aerospace engineering and biomedical implants to construction and wearable technology. A key advantage of biomimetic and bioinspired materials is their ability to achieve an optimal balance between mechanical performance and material efficiency [4]. Traditional engineering materials often suffer from trade‐offs between strength and weight, ductility and toughness, or hardness and flexibility. Nature, however, has evolved strategies to integrate these properties seamlessly. For example, nacre (mother‐of‐pearl) found in mollusk shells is composed of an intricate layering of organic and inorganic materials, providing exceptional impact resistance despite being composed primarily of brittle components. Similarly, spider silk is renowned for its incredible tensile strength and elasticity, making it one of the toughest known natural fibers [5].

The study of biomimetic materials involves a multidisciplinary approach that integrates principles from biology, chemistry, physics, and material science. By understanding the fundamental principles behind the formation of biological materials, scientists can translate these principles into engineering designs [6]. This process typically involves analyzing the microstructures of biological materials, replicating their composition through synthetic means and refining fabrication techniques to achieve the desired properties. Advanced imaging technologies, such as scanning electron microscopy (SEM) and atomic force microscopy (AFM), have played a crucial part in revealing the intricate nanoscale details of natural materials, providing valuable insights for the development of bioinspired materials. One of the most fascinating aspects of biomimetic materials is their ability to adapt to different environmental conditions [7]. Natural materials often exhibit self‐healing capabilities, responsiveness to stimuli, and efficient energy dissipation mechanisms. Inspired by these properties, scientists have created self‐repairing materials, shape‐memory polymers, and adaptive composites that can alter their mechanical behavior in response to external factors. For example, bone is a dynamic material that remodels itself continuously in response to mechanical stress. Scientists are developing bone‐mimicking materials with bioresorbable and regenerative properties for use in medical applications such as orthopedic implants and tissue engineering scaffolds [8, 9]. Moreover, biomimetic approaches are leading to the development of environmentally sustainable materials. Traditional synthetic materials, particularly plastics and composites, pose significant environmental challenges due to their non‐biodegradable nature and reliance on fossil fuels. Nature, on the other hand, has evolved materials that are biodegradable, recyclable, and produced through energy‐efficient processes [10].

Researchers are increasingly exploring bio‐based polymers, nanocellulose fibers, and bioinspired adhesives that offer sustainable alternatives to conventional materials. Another area where biomimetic materials are making a significant impact is in aerospace and automotive engineering. The aerospace industry continuously seeks lightweight materials with superior mechanical performance to enhance fuel efficiency and durability. Inspired by the lightweight yet strong structure of bird bones, engineers have designed hollow, lattice‐structured composites that reduce material weight without compromising strength. Similarly, shark skin's micro‐textured surface has inspired drag‐reducing coatings for aircraft and underwater vehicles, improving aerodynamic and hydrodynamic efficiency [11]. In construction and civil engineering, biomimetic principles are being applied to develop stronger, more durable, and self‐healing materials. Traditional concrete is prone to cracking and degradation over time, leading to costly repairs and maintenance. Inspired by the self‐healing properties of certain biological systems, researchers have developed bio‐concrete containing bacteria that produce limestone to seal cracks automatically [12]. This innovation enhances the longevity of structures and reduces the environmental impact associated with construction material production. Furthermore, biomimetic materials are transforming the field of robotics and wearable technology. Soft robotics, inspired by flexible biological organisms such as octopuses and jellyfish, utilizes bioinspired materials that enable robots to move with enhanced dexterity and adaptability. These materials provide robots with a level of flexibility and resilience that traditional rigid materials cannot achieve. Wearable technologies, including bioinspired textiles and smart materials, are being developed to enhance comfort, durability, and functionality in clothing and medical devices [13].

The development of biomimetic materials is inextricably linked to advances in manufacturing processes. Traditional production processes sometimes fail to recreate the intricate hierarchical patterns seen in natural materials. However, recent advances in additive manufacturing (3D printing), chemical vapor deposition, and self‐assembly methods have allowed for the exact manufacture of bioinspired structures. Additive manufacturing, in particular, enables the fabrication of complex geometries with specific mechanical characteristics, creating new opportunities for customized biomimetic materials in a variety of sectors [14, 15]. Despite the obvious benefits of biomimetic and bioinspired materials, some difficulties must be overcome in order to attain broad acceptance. Scalability in production is a significant hurdle. While nature manufactures materials using extremely efficient biological processes, synthesizing similar processes on an industrial scale is difficult and expensive.

Furthermore, assuring the long‐term stability and endurance of biomimetic materials in real‐world settings is an ongoing research topic. Scientists are attempting to optimize manufacturing procedures, improve material durability, and incorporate bioinspired designs into current engineering systems [16, 17]. In summary, biomimetic and bioinspired materials offer a new way of creating materials, providing unparalleled mechanical performance through nature‐inspired methods. By analyzing and simulating biological systems, engineers and scientists are creating novel materials with applications in aerospace, biomedical engineering, construction, and robotics. As research in this field progresses, multidisciplinary partnerships and technological advances will be essential for realizing the full potential of biomimetic materials. The future of engineering materials lies in harnessing nature's wisdom to find stronger, more efficient, and longer‐lasting solutions to current technological challenges [18, 19].

## 2 Design Principles of Biomimetic Materials

**FIGURE 1:**
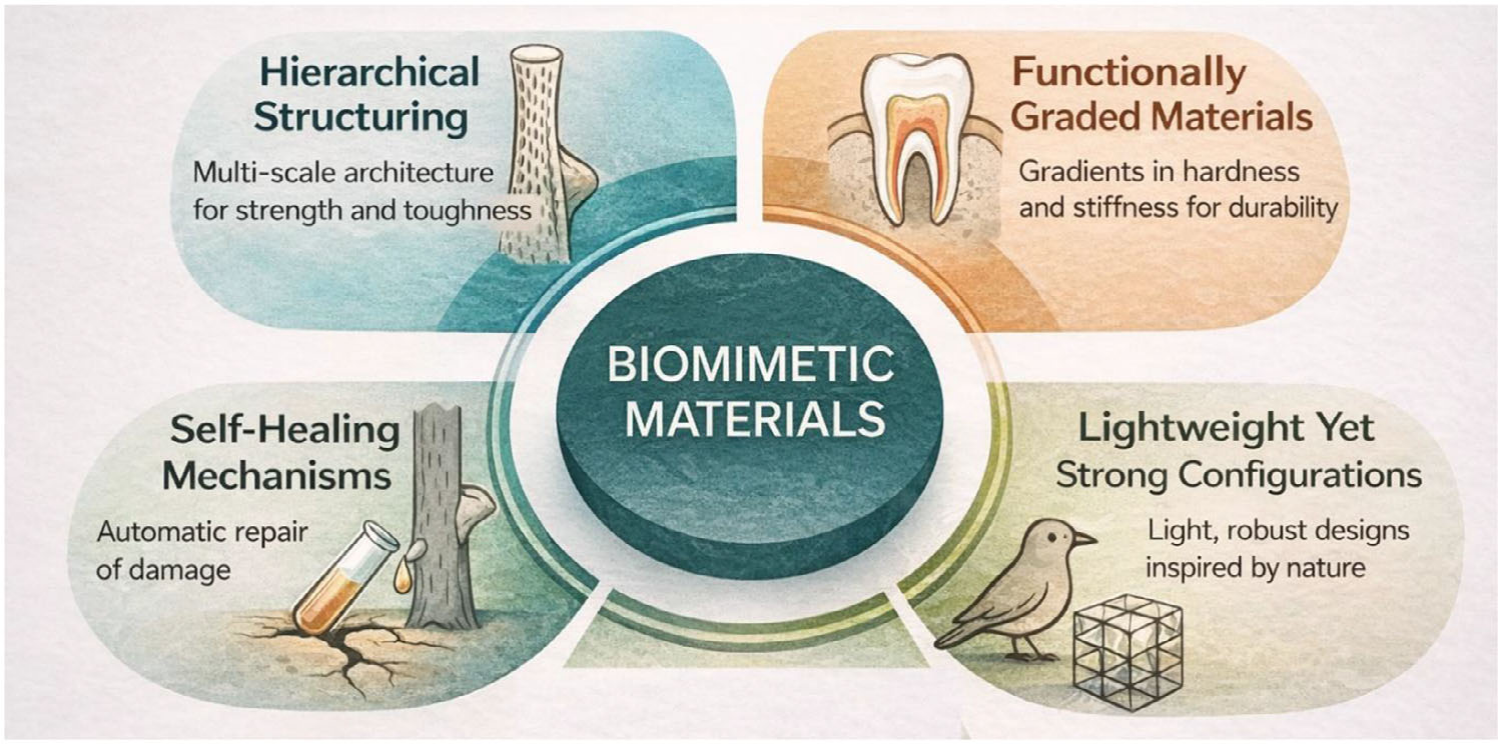
Design principles of Biomimetic Materials.

The exceptional mechanical performance of biomimetic materials, as shown in Figure 1, stems from nature's ability to create highly efficient, complex structures. By mimicking these designs, engineers and researchers can develop materials that are stronger, more resilient, and more functional. Key design principles contributing to the enhanced mechanical performance of biomimetic materials include hierarchical structuring, functionally graded materials, self‐healing mechanisms, and lightweight yet strong configurations [20].

### 2.1 Hierarchical Structuring

Hierarchical structuring enables biological materials to overcome the classical strength–toughness trade‐off observed in monolithic materials [21]. Rather than relying solely on intrinsic material strength, these systems achieve mechanical robustness through multiscale architecture.

Bone exhibits a tensile strength of approximately 100–150 MPa and a compressive strength of 130–200 MPa, while maintaining a fracture toughness in the range of 2–12 MPa·m^1/2^ depending on orientation and age [22]. These values are significantly higher than those of pure hydroxyapatite (∼1 MPa·m^1/2^ fracture toughness), demonstrating that hierarchical collagen–mineral coupling enhances crack resistance through mechanisms such as fibril pull‐out, crack deflection, and microcracking. Importantly, bone maintains a density of ∼1.8–2.0 g/cm^3^, offering a favorable strength‐to‐weight ratio compared to many engineering ceramics.

Nacre achieves a fracture toughness of approximately 5–10 MPa·m^1/2^, nearly 1000 times higher than monolithic aragonite (∼0.003–0.005 MPa·m^1/2^) [23, 24]. Its tensile strength ranges between 80 and 130 MPa despite being composed of ∼95% brittle ceramic. This performance enhancement results from controlled platelet sliding, mineral bridge formation, and viscoelastic deformation of the organic interlayer [25].

Synthetic nacre‐inspired composites have reported toughness improvements of 3–10 times compared to traditional ceramics. However, most artificial systems remain limited to small‐scale specimens (<10 cm) and often exhibit reduced stiffness due to polymer‐rich interfaces. Therefore, while hierarchical structuring demonstrably enhances toughness, scalability, and stiffness retention remain key engineering challenges.

### 2.2 Functionally Graded Materials (FGMs)

Functionally graded biological materials mitigate stress concentration by continuously varying modulus and hardness [26]. This gradient reduces interfacial delamination and enhances fatigue resistance. Human enamel exhibits a hardness of 3–5 GPa and an elastic modulus of ∼70–110 GPa, whereas dentin shows a lower modulus of 15–25 GPa and hardness of 0.5–1 GPa [27]. The gradual transition across the enamel–dentin junction reduces stress amplification and suppresses crack penetration into softer tissue. Finite element analyses show that graded interfaces can reduce peak interfacial stress by 20%–40% compared to sharp interfaces.

Bamboo demonstrates radial density variation from approximately 0.4 g/cm^3^ in the inner region to 0.9 g/cm^3^ near the outer wall, resulting in tensile strengths up to 200–400 MPa along the fiber direction [28]. This gradient optimizes bending stiffness while minimizing weight.

In engineered systems, metal–ceramic FGMs used in thermal barrier coatings can withstand temperature gradients exceeding 1000°C while maintaining structural integrity [28]. In biomedical implants, graded porosity (30%–70%) reduces elastic modulus from ∼110 GPa (solid titanium) to 10–30 GPa, closer to cortical bone (∼20 GPa), thereby mitigating stress shielding. Despite these advantages, most synthetic FGMs rely on stepwise layering rather than continuous gradients, limiting their ability to replicate biological stress redistribution efficiency [29].

### 2.3 Self‐Healing Mechanisms

Self‐healing is a fundamental property found in many biological materials, enabling them to repair damage and restore their mechanical integrity. This ability is particularly important for materials that undergo repetitive mechanical stress, environmental degradation, and wear over time. Scientists have discovered synthetic materials that can mend damage and fractures independently, thereby prolonging service life and reducing maintenance costs [30]. Tree bark is a typical example of a natural self‐healing mechanism. When a tree is injured, it secretes resin or sap, which rapidly closes the wound and prevents infections and more harm. Inspired by this technique, researchers created self‐healing polymers with microcapsules containing healing chemicals. When the material is damaged, the capsules break open and release their contents, filling the fractures and restoring the material's mechanical properties [31]. Seashells also have self‐healing properties. When tiny fractures appear, biological processes help deposit calcium carbonate at the damaged spot, eventually repairing the structure. This process has been replicated in the development of bioinspired self‐healing concrete, which incorporates bacteria that produce limestone to repair fractures [32]. This invention dramatically improves the durability and sustainability of infrastructure while reducing maintenance costs. Self‐healing materials are being developed for use in electronics and coatings, including anti‐corrosion coatings, protective films, and flexible electronics. These materials help to prevent failures caused by environmental stress and mechanical wear, thereby improving reliability in sectors such as aerospace, automotive, and healthcare [33]. Biological self‐healing systems actively restore structural integrity through cellular processes. In contrast, synthetic analogues are typically passive and limited in healing cycles.

Microcapsule‐based self‐healing polymers can recover 60%–90% of their original fracture toughness after a single crack event [31]. However, repeated damage reduces healing efficiency due to depletion of encapsulated agents. Bacteria‐based self‐healing concrete systems inspired by biomineralization can seal cracks up to 0.5 mm in width and restore up to 80%–100% of water tightness [32]. Compressive strength recovery typically ranges between 15% and 30% after healing cycles, depending on environmental conditions. Intrinsic self‐healing polymers based on reversible covalent bonding demonstrate healing efficiencies exceeding 90% tensile strength recovery under controlled laboratory conditions [33]. However, healing often requires elevated temperatures or extended times (12–48 h), limiting immediate structural applications. For aerospace‐grade composites, where tensile strengths exceed 500–1500 MPa (carbon fiber composites), integrating healing mechanisms without compromising stiffness remains a major challenge. Current self‐healing systems often reduce baseline mechanical properties by 5%–20% due to added inclusions or soft phases.

### 2.4 Lightweight yet Strong Configurations

One of nature's greatest engineering achievements is its ability to create materials that are lightweight yet incredibly strong. Many biological materials achieve this balance through the use of porous structures, optimized load‐bearing configurations, and efficient material distribution. This principle is crucial for applications where reducing weight is essential, such as in the aerospace, automotive, and sports equipment industries. [34, 35]. Bird bones are a prime example of lightweight yet strong structures. Unlike the dense, solid bones of mammals, bird bones are hollow with internal struts and supports that maximize strength while minimizing weight. Bird bones exhibit densities as low as 0.6–0.8 g/cm^3^ while maintaining compressive strengths sufficient to support dynamic flight loads [36]. Their hollow tubular geometry with internal struts enhances buckling resistance without significant mass increase. Spider silk exhibits tensile strengths of 1–1.5 GPa and extensibility up to 30%, resulting in toughness values approaching 150–200 MJ/m^3^ [37, 38]. By comparison, high‐strength steel has tensile strength of ∼1–2 GPa but extensibility below 10%, yielding significantly lower toughness per unit weight. Additively manufactured lattice structures inspired by trabecular bone can achieve weight reductions of 40%–70% compared to solid components while retaining 60%–90% of stiffness [39]. However, fatigue resistance and defect sensitivity remain concerns for large‐scale deployment. Therefore, while lightweight bioinspired configurations demonstrate clear strength‐to‐weight advantages, translating laboratory‐scale architectures into defect‐tolerant industrial components remains an open research area.

The ideas of hierarchical structure, functionally graded materials, self‐healing mechanisms, and lightweight yet robust configurations have transformed material design in engineering. Biomimetic materials have exhibited greater mechanical performance by replicating nature's highly optimized techniques, allowing for advances in aerospace, biomedical engineering, building, and a variety of other sectors [40]. Ongoing research and technical breakthroughs are refining these bioinspired techniques and opening the door to next‐generation materials with unique properties. As cooperation between material scientists, biologists and engineers expands across disciplines, the ability of biomimetic materials to address complex engineering challenges becomes increasingly promising. The future of materials science lies in leveraging nature's inventiveness to create sustainable, high‐performance materials for a wide range of applications [41].

## 3 Bioinspired Materials

**FIGURE 2:**
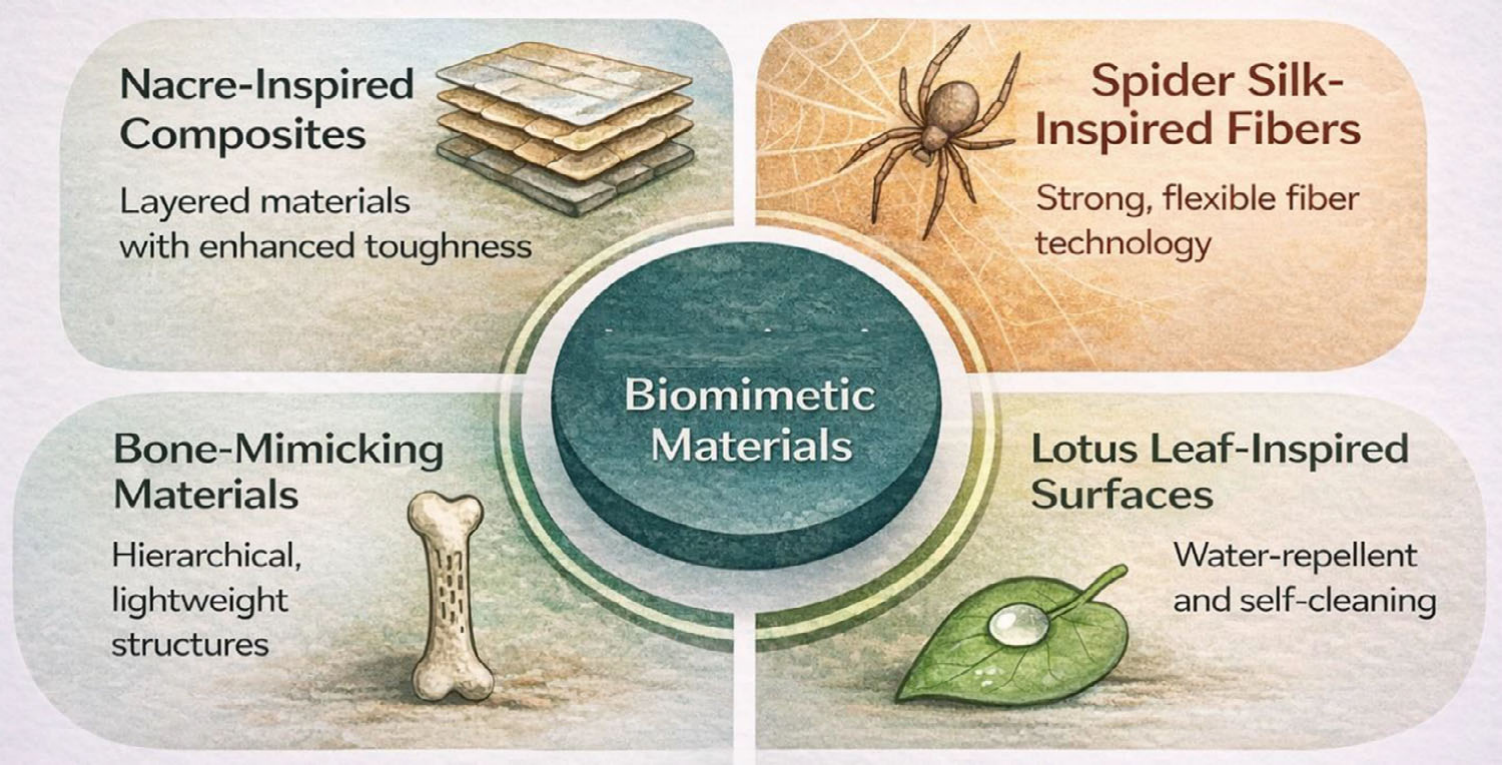
Bioinspired Materials.

The study of biomimetic materials has led to various synthetic materials replicating natural structures' exceptional mechanical properties. Promising bioinspired materials include nacre‐inspired composites, spider silk‐inspired fibres, bone‐mimicking materials, and lotus leaf‐inspired surfaces, as shown in Figure 2. These materials offer innovative solutions for a wide range of technical applications, including in the fields of aerospace and biomedical engineering, as well as for improved coatings and textiles [42].

### 3.1 Nacre‐Inspired Composites

Nacre, also known as mother‐of‐pearl, is a highly tough and impact‐resistant material found in mollusk shells. Its exceptional mechanical properties arise from its unique layered structure, where aragonite platelets are bonded together by a biopolymer matrix. This brick‐and‐mortar arrangement allows nacre to effectively dissipate energy and prevent catastrophic failure [43]. Synthetic nacre‐inspired composites have demonstrated fracture toughness values ranging from 3–15 MPa·m^1/2^, compared to 1–3 MPa·m^1/2^ for conventional structural ceramics [43, 44]. Impact resistance improvements of 2–5 times have been reported relative to monolithic ceramic systems. However, tensile strengths of these bioinspired composites (100–300 MPa) remain lower than advanced fiber‐reinforced composites (>800 MPa). Therefore, their optimal application may lie in impact‐resistant panels and coatings rather than primary load‐bearing aerospace structures.

Scientists have successfully replicated the layered structure of nacre using synthetic composites of ceramics and polymers. These nacre‐like materials are significantly tougher and more resistant to impact than conventional engineering materials. By incorporating nanoscale layering techniques, researchers have enhanced the mechanical performance of these composites for use in protective coatings, aerospace components and lightweight armor systems [44]. The ability to combine high strength with lightweight properties makes nacre‐inspired composites an attractive option for next‐generation structural materials.

### 3.2 Spider Silk‐Inspired Fibers

Spider silk is noted for its exceptional tensile strength and suppleness, making it one of the most durable natural fibres. Spider silk's molecular structure comprises well‐organized protein chains that create strong hydrogen bonds, which help explain its outstanding mechanical capabilities. Despite its small weight, spider silk outperforms steel in strength and Kevlar in toughness, making it a highly sought‐after biomimetic material [45, 46]. Recombinant spider silk fibers typically achieve tensile strengths of 0.5–1.2 GPa and elongation at break of 10%–25% [45, 47]. Although promising, these values still fall below natural spider silk performance due to incomplete molecular alignment. Scientists have created synthetic silk‐like fibres by combining recombinant protein engineering, electrospinning, and biofabrication processes. Researchers can create recombinant spider silk proteins by genetically altering bacteria, yeast, and even plants. These bioinspired fibres have applications in textiles, biomedicine, and aeronautical engineering. Spider silk‐based sutures and scaffolds are highly biocompatible, making them ideal for wound healing and tissue engineering. Furthermore, its lightweight but high‐strength qualities make it an excellent choice for high‐performance textiles and sophisticated structural materials in aerospace [47, 48].

### 3.3 Bone‐Mimicking Materials

Bone is a remarkable biological material that reaches an ideal combination of strength, toughness, and lightness due to its hierarchical porosity structure. The combination of collagen and hydroxyapatite minerals gives bone toughness and resilience, enabling it to sustain high mechanical loads while constantly remodeling to respond to environmental pressures [46, 48]. Additively manufactured bone scaffolds with porosity levels between 50% and 80% exhibit compressive strengths ranging from 2–50 MPa, comparable to cancellous bone (2–12 MPa) [46, 48, 49]. Elastic modulus values can be tuned between 0.5 and 20 GPa depending on porosity. Researchers used additive manufacturing, biomineralization, and biofabrication methods to create bioinspired bone‐like materials based on bone architecture. These materials are very valuable in biomedical applications such orthopedic implants, bone graft replacements, and scaffolds for tissue engineering. Additive manufacturing (3D printing) allows for the exact construction of porous structures that resemble the natural bone matrix, facilitating cell proliferation and integration with surrounding tissue [49, 50]. These bone‐mimicking materials, which include bioactive minerals such as calcium phosphate‐based compounds, may improve osseointegration and expedite bone repair, revolutionizing the area of regenerative medicine.

### 3.4 Lotus Leaf‐Inspired Surfaces

The lotus leaf has remarkable superhydrophobic properties, allowing water droplets to roll off its surface carrying dirt particles away with them. This self‐cleaning ability, known as the “lotus effect,” is attributed to the micro‐ and nanoscale roughness of the leaf surface combined with a hydrophobic wax coating [51, 52]. Lotus‐inspired superhydrophobic coatings typically exhibit water contact angles exceeding 150° and sliding angles below 10°. Anti‐corrosion coatings based on such surfaces have demonstrated corrosion current reductions of one to two orders of magnitude compared to untreated steel [50].

Nevertheless, abrasion resistance remains a limitation. Many coatings lose superhydrophobicity after 100–1000 abrasion cycles under laboratory testing, highlighting the need for mechanically robust surface architectures [53].

Inspired by this natural phenomenon, engineers have developed self‐cleaning and anti‐fouling surfaces that mimic the lotus leaf's structure. These surfaces have widespread applications in engineering, including waterproof coatings, anti‐corrosion treatments, and stain‐resistant textiles. Superhydrophobic coatings are particularly valuable in industries such as construction, automotive, and aerospace, where reducing maintenance and enhancing durability are critical. Additionally, biomedical applications of lotus leaf‐inspired materials include anti‐bacterial coatings for medical devices, ensuring hygiene and reducing the risk of infections [54].

**FIGURE 3:**
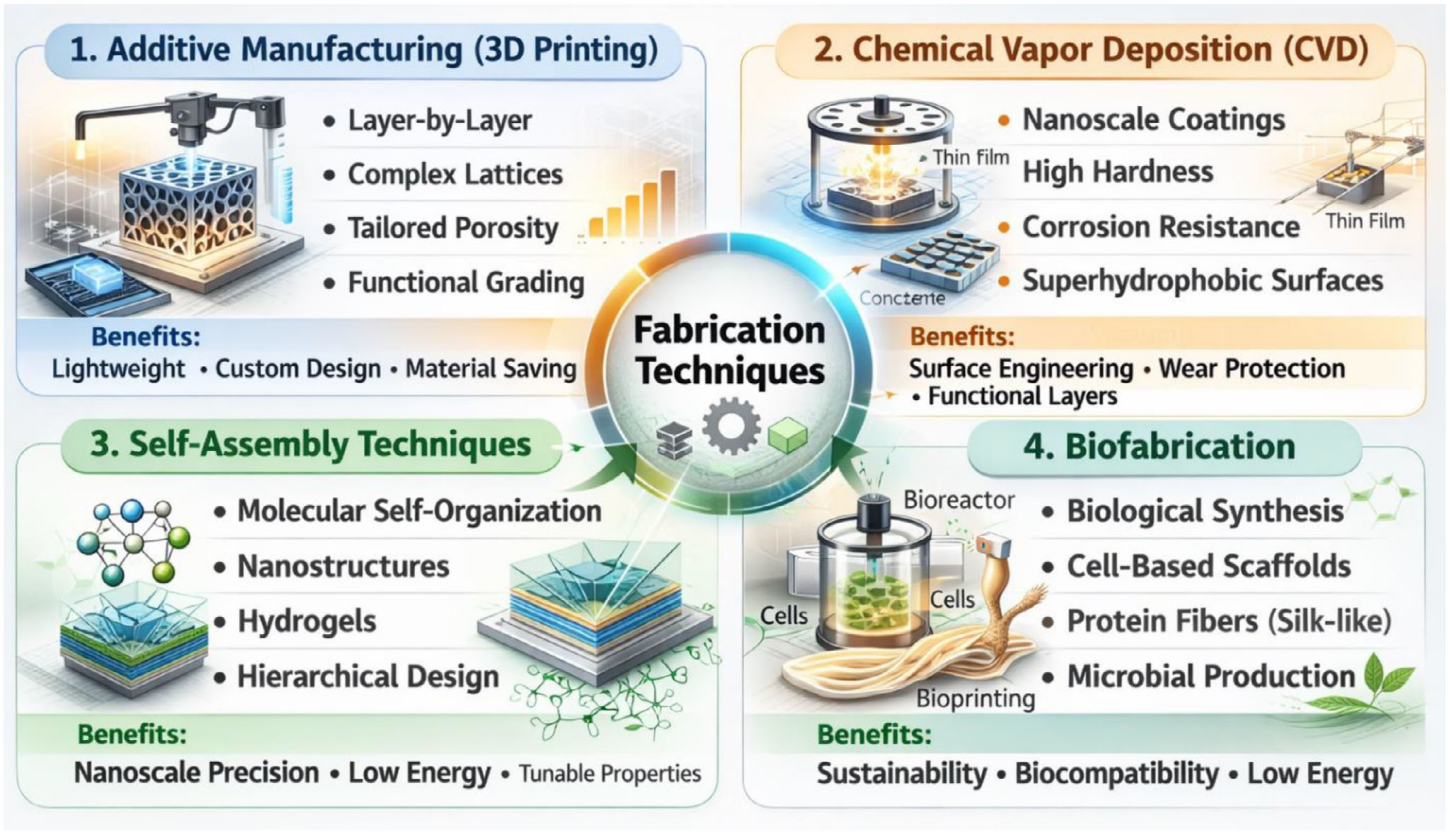
Fabrication Techniques.

The development of bioinspired materials has opened new frontiers in material science and engineering. By emulating the highly efficient designs found in nature, scientists have created materials with superior mechanical properties, adaptability, and functionality. Nacre‐inspired composites, spider silk‐inspired fibers, bone‐mimicking materials, and lotus leaf‐inspired surfaces are just a few examples of how biomimetic principles revolutionize industries ranging from aerospace and biomedicine to textiles and construction. As research and fabrication techniques (Figure 3) continue to advance, the potential for bioinspired materials to drive innovation and sustainability in engineering applications remains vast [54].

## 4 Fabrication Techniques for Bioinspired Materials

Bioinspired materials are developed using sophisticated manufacturing processes that allow for exact control over structure, content, and usefulness. These methods are critical for reproducing nature's complicated patterns and improving mechanical performance. The most successful fabrication methods include additive manufacturing (3D printing), chemical vapor deposition (CVD), self‐assembly approaches, and biofabrication [52, 55].

### 4.1 Additive Manufacturing (3D Printing)

Additive manufacturing (AM) enables the spatially controlled, layer‐by‐layer deposition of materials, allowing the fabrication of architected and functionally graded structures that are difficult or impossible to achieve through conventional subtractive or molding processes [56, 57]. Unlike traditional manufacturing routes, AM permits precise control over internal architecture, including tailored porosity ranging from approximately 10%–90%, gradient material distribution, complex lattice geometries, and multi‐material integration within a single build. This level of design freedom is particularly significant for biomimetic systems, where hierarchical structuring and spatial variation in properties govern mechanical performance.

Through manipulation of geometric parameters such as strut thickness, unit cell topology, and pore size, AM enables tuning of elastic modulus across a broad range (approximately 0.5–30 GPa depending on architecture and material selection) [58]. Controlled pore sizes, typically between 100 and 500 µm in porous constructs, allow systematic modulation of stiffness, strength, and transport properties. Furthermore, topology‐optimized lattice structures can achieve mass reductions of 30%–70% while retaining 60%–90% of the stiffness of fully dense counterparts [59], demonstrating the efficiency of geometry‐driven reinforcement. Importantly, AM supports continuous functional grading and multi‐scale architectural control—capabilities not attainable through conventional lamination or monolithic processing techniques. Thus, additive manufacturing functions as a platform technology for implementing hierarchical organization, functional grading, and lightweight structural design within engineered materials [60].

### 4.2 Chemical Vapor Deposition (CVD)

Chemical vapor deposition (CVD) enables nanoscale coating deposition with thickness control ranging from a few nanometers to several micrometers through the controlled reaction of gaseous precursors on a substrate surface [61]. In contrast to additive manufacturing, which primarily defines bulk architecture, CVD is a surface‐engineering technique that tailors interfacial and surface‐dominated properties without significantly altering the underlying structural geometry.

CVD‐produced coatings substantially enhance surface performance metrics. Hard ceramic coatings fabricated via CVD can achieve hardness values exceeding 20 GPa, improving wear resistance and extending service life under abrasive conditions. Corrosion resistance can improve by one or more orders of magnitude, as reflected by significant reductions in corrosion current density. Superhydrophobic surfaces engineered through nanoscale texturing and low‐surface‐energy coatings can exhibit water contact angles greater than 150°, while maintaining improved coating uniformity and adhesion [62]. Additionally, graphene synthesized via CVD demonstrates intrinsic tensile strengths exceeding 100 GPa and exceptional electrical conductivity, enabling multifunctional surfaces with both mechanical robustness and electronic performance [52].

Overall, CVD contributes primarily to surface‐dominated mechanical, chemical, and functional enhancement, complementing bulk fabrication strategies by providing nanoscale control over interfacial behavior and surface performance.

### 4.3 Self‐Assembly Techniques

Self‐assembly represents a bottom‐up fabrication strategy driven by thermodynamic minimization and governed by molecular‐scale interactions such as hydrogen bonding, electrostatic attraction, and van der Waals forces [63]. Unlike top‐down manufacturing methods, self‐assembly enables spontaneous organization of molecules, nanoparticles, or macromolecular building blocks into ordered structures with high nanoscale precision. This approach is particularly effective for controlling interfacial structure, periodicity, and hierarchical organization at length scales typically below several hundred nanometers.

Through these interactions, self‐assembly facilitates the formation of layered nanocomposites, peptide‐based hydrogels, photonic crystal architectures, and nanostructured barrier films. Layered self‐assembled composites have demonstrated fracture toughness improvements of approximately 3–5 times compared to brittle ceramic counterparts, primarily due to controlled interfacial sliding and crack deflection mechanisms [64]. Peptide‐based hydrogels exhibit tunable elastic moduli in the range of 0.1–100 kPa, allowing precise adjustment of mechanical stiffness through molecular design while maintaining biocompatibility and predictable degradation behavior [65, 66]. In addition, periodic nanoscale ordering achieved through self‐assembly enables photonic materials capable of manipulating light via constructive interference, resulting in anti‐reflective surfaces and structural coloration effects [67]. Compared to additive manufacturing and chemical vapor deposition, self‐assembly is most advantageous for nanoscale precision and interfacial engineering rather than for macroscale load‐bearing architectures, making it particularly valuable in multifunctional and hybrid material systems [68].

### 4.4 Biofabrication

Biofabrication integrates principles from synthetic biology, tissue engineering, and materials science to generate materials through biologically mediated synthesis pathways rather than conventional thermomechanical processing [69, 70, 71]. Instead of relying solely on external shaping and consolidation, biofabrication leverages cellular machinery and biochemical reactions to construct functional materials under mild processing conditions. Core strategies include recombinant protein production, microbial fermentation, bioprinting of living cells, and enzymatic mineralization.

Recombinant protein engineering enables the production of synthetic silk‐like fibers with tensile strengths in the range of 0.5–1.2 GPa, approaching the lower bound of natural spider silk performance [72]. Bioprinting allows spatially controlled deposition of cells and biomaterials to create organized, multicellular constructs, including vascularized scaffolds designed to improve nutrient transport and structural integration [73]. In parallel, microbial fermentation processes produce bacterial cellulose with tensile strengths of approximately 200–300 MPa and high crystallinity, offering excellent purity and mechanical stability for advanced biomaterials applications [74].

Biofabrication presents sustainability advantages, including lower processing temperatures, reduced energy consumption, and reliance on renewable biological feedstocks. However, significant challenges remain in scaling production, ensuring batch‐to‐batch reproducibility, maintaining long‐term mechanical stability, and navigating regulatory approval pathways for biologically derived materials [75].

## 5 Challenges in the Development of Bioinspired Materials

Despite their demonstrated performance advantages, bioinspired materials face significant translational barriers that limit widespread industrial adoption. Current challenges extend beyond general issues of cost and fabrication; they involve multi‐scale manufacturing control, reproducibility, lifecycle durability, qualification standards, and supply‐chain scalability. Addressing these constraints requires not only materials innovation but also process engineering, digital manufacturing integration, and performance standardization frameworks [76, 77].

### 5.1 Manufacturing Complexity and Scalability

**FIGURE 4:**
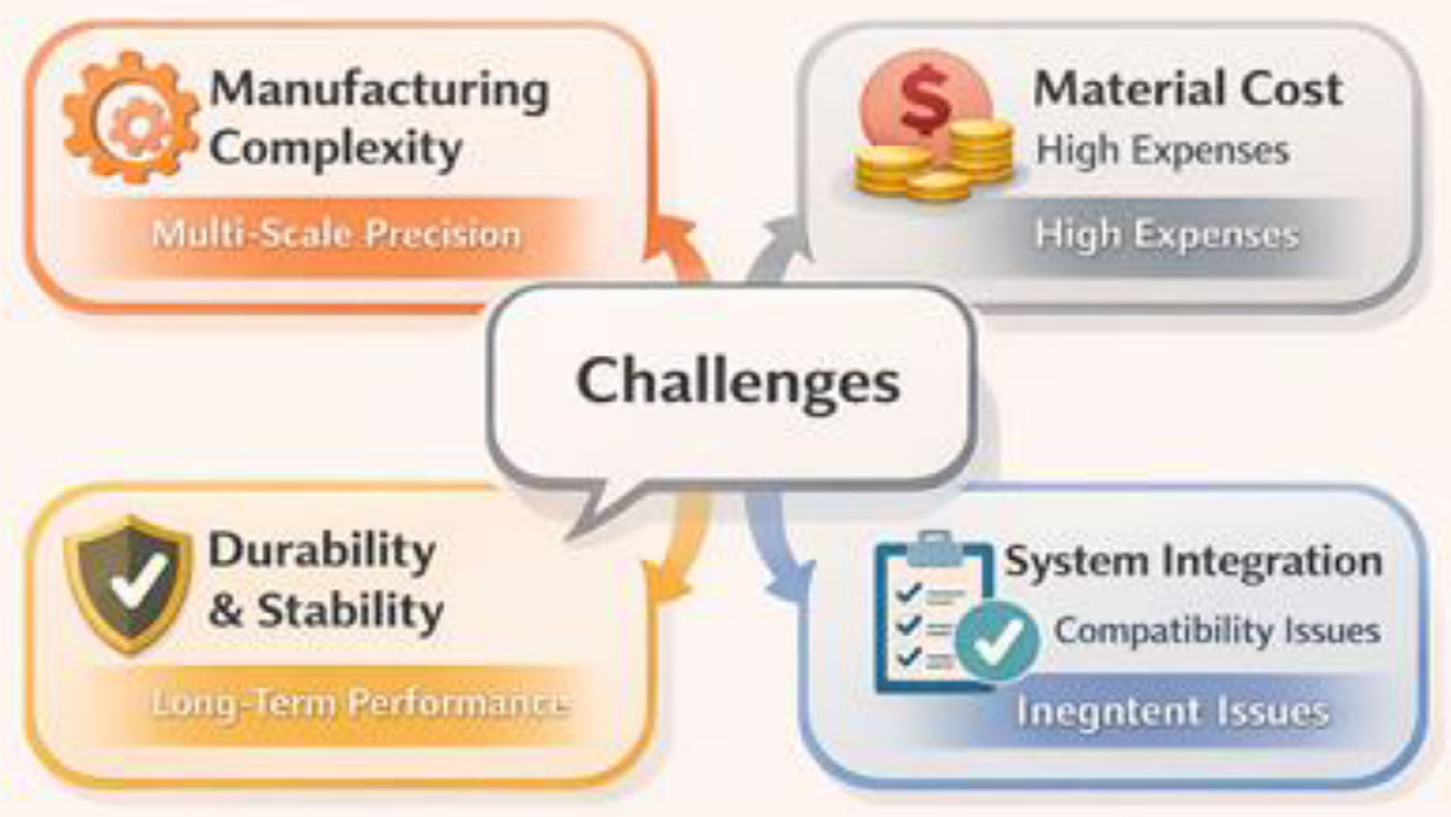
Challenges in Developing Bioinspired Materials.

As shown in Figure 4, A central technical challenge lies in replicating hierarchical architectures spanning nano‐, micro‐, and macro‐scales. Many bioinspired materials derive their superior toughness and damage tolerance from precisely organized interfaces, graded compositions, or architected porosity. Conventional high‐throughput methods such as injection moulding, extrusion, or compression moulding are not designed to control structural features across multiple length scales simultaneously [46, 78]. Advanced additive manufacturing (e.g., high‐resolution 3D printing and two‐photon polymerization) enables geometric precision but remains constrained by build rate, anisotropy, material compatibility, and process repeatability [79]. Laboratory‐scale demonstrations often achieve excellent structural fidelity, yet translating these processes to industrial volumes introduces defects, dimensional variability, and inconsistent interfacial bonding. Self‐assembly and biofabrication approaches—such as microbial synthesis or biomineralization—offer bottom‐up fabrication routes with inherent hierarchical control. However, these methods require tightly regulated environmental conditions and exhibit sensitivity to temperature, pH, nutrient supply, and contamination. Production speed, batch‐to‐batch reproducibility, and downstream processing remain bottlenecks for commercialization [80]. Scalability therefore, represents a systems‐level engineering challenge: it requires automation, inline quality monitoring, digital process control, and hybrid manufacturing strategies that combine conventional forming with architected design. Emerging approaches such as 4D printing introduce adaptive functionality but further increase process complexity and validation requirements [81, 82].

### 5.2 Material Cost

Cost remains a critical adoption barrier, particularly in sectors such as construction and transportation, where margins are narrow. Bioinspired materials frequently rely on specialized feedstocks (engineered proteins, nanomaterials, bio‐derived polymers) and energy‐intensive fabrication routes. Microbial production of recombinant proteins, for example, requires controlled fermentation, purification, and post‐processing steps that elevate operational expenses [83].

Beyond raw material costs, the economic burden includes extended R&D cycles, pilot‐scale validation, tooling redesign, workforce training, and regulatory testing. Many promising systems remain confined to laboratory‐scale batches due to low production yield and high capital investment requirements for scale‐up [84].

A forward‐looking cost strategy must therefore include: (i) use of abundant or waste‐derived bioresources, (ii) yield optimization in fermentation and biofabrication, (iii) automation and AI‐assisted process optimization, and (iv) design‐for‐manufacturability principles introduced early in material development [85, 86]. Without parallel advances in manufacturing efficiency, performance gains alone will not justify industrial transition.

#### 5.2.1 Strategies for Cost Reduction

1. Developing Cost‐Effective Synthesis Techniques – Utilizing abundant and renewable raw materials, such as plant‐based polymers or waste‐derived biomaterials, can reduce dependence on expensive engineered components.
2. Optimizing Microbial and Plant‐Based Biofabrication – Enhancing fermentation efficiency and genetic engineering techniques can improve yield while reducing resource consumption.
3. Enhancing Production Efficiency – Integrating automation, AI‐driven material optimization, and streamlined processing methods can lower operational costs.
4. Exploring Alternative Biomimetic Formulations – Investigating naturally occurring bioinspired structures that require minimal modification for industrial use.
5. Encouraging Industrial Adoption through Incentives – Providing subsidies, tax incentives, and funding support for bioinspired material development can lower economic barriers to entry [87].

### 5.3 Durability and Stability

Although natural materials such as nacre or spider silk exhibit exceptional toughness, their biological counterparts are not inherently optimized for long‐term exposure to UV radiation, moisture cycling, chemical attack, or elevated temperatures. Synthetic bioinspired analogues must therefore demonstrate not only high initial performance but stable properties over extended service lifetimes [88]. Key durability concerns include hydrolytic degradation, creep under sustained loading, interfacial debonding in layered architectures, and fatigue‐driven microcracking. In infrastructure and aerospace applications, even minor degradation mechanisms can accumulate over decades, compromising reliability. Current research focuses on crosslinking strategies, protective coatings, hybrid organic–inorganic reinforcement, and interface engineering to improve environmental resistance while retaining hierarchical toughening mechanisms. However, accelerated aging data, long‐term field validation, and standardized durability benchmarks remain limited. Lifecycle assessment frameworks must also account for repairability and recyclability to ensure sustainability claims are substantiated.

### 5.4 Integration Into Existing Systems

Even when technical performance is validated, integration into existing industrial ecosystems presents additional barriers. Manufacturing lines, tooling systems, certification standards, and supply chains are optimized for conventional materials. Introducing bioinspired alternatives often necessitates requalification testing, process modification, and compliance with established structural or biomedical regulations [89]. In aerospace and biomedical sectors particularly, certification pathways require extensive mechanical testing, fatigue validation, biocompatibility assessment, and long‐term reliability studies. These processes are time‐consuming and capital intensive, slowing adoption despite promising laboratory results. Future progress depends on establishing standardized testing protocols tailored to architected and hierarchical materials, digital twins for predictive performance modeling, and cross‐disciplinary collaboration between material scientists, process engineers, and regulatory bodies [90].

The key challenges—manufacturing accuracy, scalability, cost, durability, and system integration—are interconnected. Addressing them requires advances in automated production, improved material design, sustainable raw materials, and lifecycle assessment. To achieve widespread adoption, bioinspired materials must move beyond laboratory research and become manufacturable, reliable, certifiable, and cost‐effective for industrial use.

## 6 Applications of Bioinspired Materials

**FIGURE 5:**
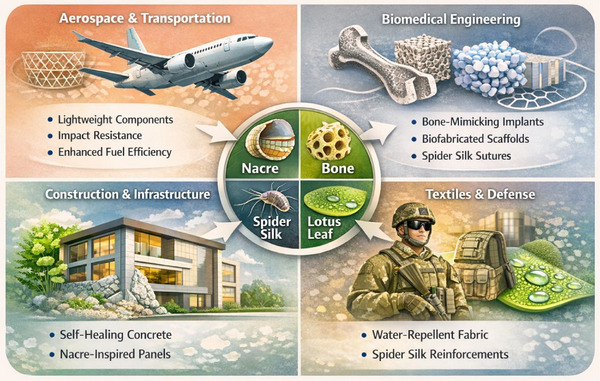
Applications of Bioinspired Materials.

Bioinspired materials have been used in various applications (Figure 5) due to their superior mechanical qualities, flexibility, and sustainability. From aerospace to biomedical engineering, these materials provide unique solutions that outperform traditional materials in terms of strength, durability, and usefulness [91, 92].

### 6.1 Aerospace

In aerospace systems, structural weight directly influences fuel consumption and emissions, making lightweight materials essential for improved efficiency and reduced lifecycle costs. Bioinspired architected composites and graded sandwich structures achieve 20%–50% mass reduction while maintaining stiffness and load‐bearing capacity [93, 94]. Their hierarchical designs enhance specific stiffness, energy absorption, and optimized load transfer. These materials also improve impact resistance, crack deflection, and fatigue life under cyclic loading. Surface‐engineered coatings increase corrosion resistance in harsh environments, while multilayer architectures enhance thermal stability. High fatigue resistance, impact mitigation, environmental durability, and weight optimization collectively drive their adoption in the aerospace and transportation sectors.

### 6.2 Biomedical Engineering

Biomedical applications demand simultaneous mechanical compatibility and biological integration, as implants and scaffolds must closely match the mechanical properties of surrounding tissues to prevent stress shielding and premature failure. Porous scaffolds produced through additive manufacturing exhibit compressive strengths of 2–50 MPa, comparable to cancellous bone, while architectural control enables elastic moduli to be tailored toward 20–30 GPa to approximate cortical bone [95]. Optimized pore structures enhance vascularization and nutrient transport, directly linking structural design to biological performance. Additionally, biofabricated protein‐based fibers and hydrogels provide programmable degradation, high toughness for ligament replacements, tailored bioactivity in scaffolds, and effective wound healing support. In this field, success is defined not only by mechanical strength but by biocompatibility, bioresorbability, and the capacity to actively promote tissue regeneration.

### 6.3 Construction and Infrastructure

Infrastructure systems require long‐term durability under mechanical, thermal, and environmental stressors, and bioinspired materials significantly enhance structural resilience and lifecycle performance. Self‐healing concrete can autonomously seal cracks up to 0.5 mm wide and restore water tightness nearly to 100% under optimal conditions [96], thereby limiting moisture and chloride ingress, reducing reinforcement corrosion, and extending service life. Architected and layered composite panels further improve impact resistance and energy dissipation in façades, bridges, and protective structures. Through hierarchical crack deflection and interfacial sliding mechanisms, these materials offer superior toughness compared to brittle monolithic systems, resulting in extended durability, reduced maintenance costs, improved crack resistance, and greater resilience under dynamic loading.

### 6.4 Textiles and Defense

Textile and defense applications demand materials with high toughness‐to‐density ratios, flexibility, and strong environmental resistance. Bioinspired high‐performance fibers, particularly recombinant silk‐inspired systems, provide exceptional toughness relative to their weight, making them ideal for ballistic and impact‐resistant textiles [97]. Their capacity to sustain large strains before failure enhances energy absorption under dynamic loading. Surface‐functional coatings further improve performance; superhydrophobic treatments minimize moisture uptake and contamination while maintaining mechanical integrity in harsh environments [98]. Abrasion‐resistant and multifunctional coatings enhance durability in tactical and outdoor gear without compromising flexibility or comfort. Consequently, these applications focus on lightweight protection, efficient energy absorption, and resistance to environmental degradation.

## 7 Future Directions in Biomimetic and Bioinspired Materials

**FIGURE 6:**
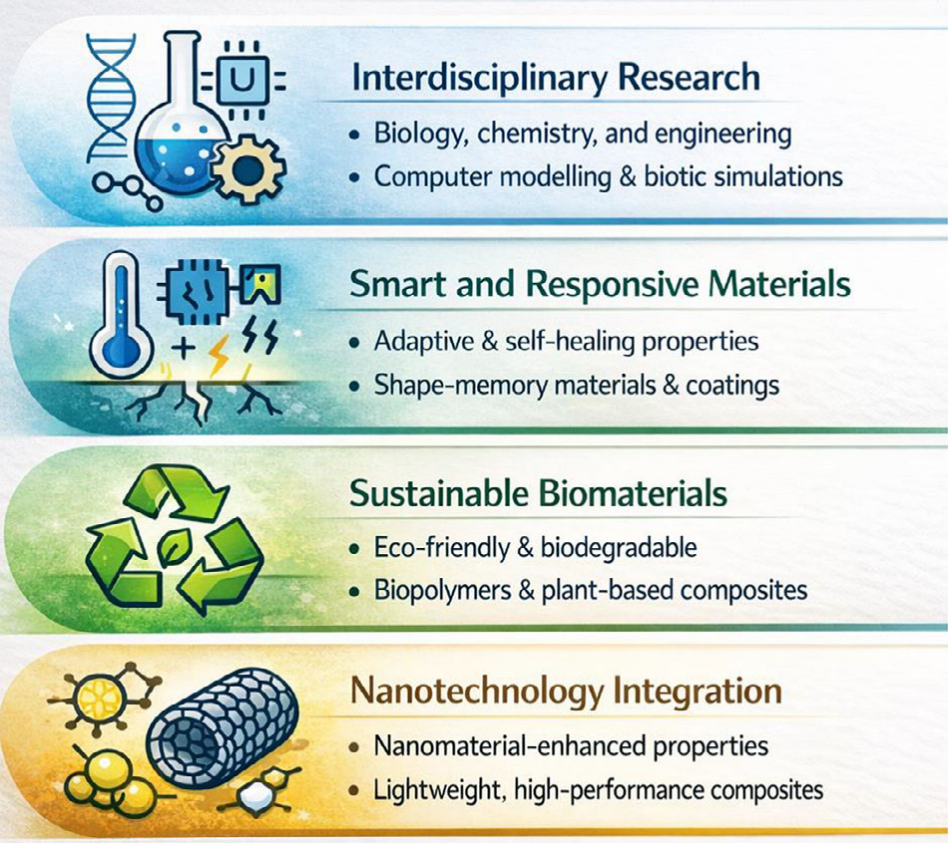
Future Directions in Biomimetic and Bioinspired Materials.

As research into biomimetic and bioinspired materials continues, many major topics emerge that will define the future materials generation. By combining multidisciplinary research, smart materials, sustainable biomaterials, and nanotechnology, bioinspired materials, as shown in Figure 6, will continue to grow with improved mechanical qualities and usefulness [99].

### 7.1 Interdisciplinary Research

Creating sophisticated biomimetic materials necessitates cooperation across several scientific fields, including biology, chemistry, material science, and engineering. Researchers may create novel materials that mimic nature's efficiency by examining the structural and functional properties of natural materials at various sizes [100]. Advances in computer modeling, biomineralization processes, and synthetic biology have increased the capacity to manufacture highly optimized bioinspired materials.

### 7.2 Smart and Responsive Materials

Inspired by biological creatures, the next generation of bioinspired materials will have adaptive and self‐healing properties. Smart materials may adapt to environmental stimuli like temperature, moisture, or mechanical stress, enabling them to alter characteristics in real time. Examples include self‐healing polymers that fix microcracks independently, shape‐memory materials that return to a preset shape, and bio‐responsive coatings that adapt to environmental circumstances. These advancements will have applications in aircraft, healthcare, and robotics [101].

### 7.3 Sustainable Biomaterials

The focus on sustainability has sparked an increased interest in eco‐friendly and biodegradable bioinspired materials. Researchers are creating natural‐source materials, such as biopolymers and plant‐based composites, to lessen environmental impact [2, 3]. Sustainable biomaterials have outstanding mechanical qualities and are also recyclable and produce little waste [102]. These materials are essential in packaging, building, and biomedical applications, where lowering carbon footprints is a goal.

### 7.4 Nanotechnology Integration

Nanotechnology plays a vital role in improving the characteristics of bioinspired materials. Researchers may increase strength, flexibility, and conductivity by integrating nanomaterials like carbon nanotubes, graphene, and nanoscale biomimetic coatings [2, 3]. Molecular‐level engineering enables precise control of material structures, resulting in lightweight, high‐performance composites for aerospace, electronics, and medical applications. The combination of nanotechnology and biomimetic design creates new possibilities for ultra‐strong, self‐assembling, and multifunctional materials [103, 104].

The future of bioinspired materials depends on the seamless integration of multidisciplinary research, smart materials, sustainability, and nanotechnology. By leveraging these developments, researchers and engineers may create materials that not only replicate nature's creativity but also outperform traditional materials in terms of performance and flexibility [105, 106]. As these technologies progress, bioinspired materials will continue to transform sectors from aerospace and healthcare to building and environmental sustainability.

## 8 Conclusion

Biomimetic and bioinspired materials provide unique prospects to improve mechanical performance in various sectors. Drawing on natural design principles, these materials achieve excellent strength, toughness, flexibility, and lightness. Despite manufacturing and scaling limitations, further advances in material science and engineering are likely to realize their full potential. This chapter explores biomimetic materials' synthesis, mechanical properties, and applications, guiding researchers and engineers toward creative and sustainable solutions. Bioinspired materials have transformed materials science by offering solutions that emulate the efficiency and flexibility of biological structures. Researchers have created materials with enhanced mechanical performance, durability, and multifunctionality by understanding and reproducing biological processes. These materials are changing industry norms in various fields, including aerospace, biomedicine, construction, and textiles. Modern production processes, such as additive manufacturing and nanotechnology, have increased the possibilities of bioinspired materials. Manufacturing complexity, cost, and long‐term stability remain challenges, but continued research and multidisciplinary cooperation are helping to overcome them.

In the future, bioinspired materials will be sustainable, innovative, and adaptable, responding to environmental stimuli and improving efficiency across various applications. As research advances, bioinspired materials will continue to drive technological developments by providing solutions that are not only high‐performing but also ecologically responsible and sustainable. In summary, bioinspired materials bridge the gap between nature and engineering, paving the way for the next generation of high‐performance, environmentally friendly, and robust materials that will define the future of science and industry.

## Funding

The authors extend their appreciation to the Deanship of Research and Graduate Studies at King Khalid University for funding this work through the Large Research Project under grant number RGP2/521/46.

## Ethics Statement

The authors have nothing to report.

## Consent

All authors have read and agreed to the published version of the manuscript.

## Conflicts of Interest

The authors declare no conflicts of interest.

## Data Availability

The data that supports the findings of this study are available in the supplementary material of this article.

## References

[1] R. R. Naik and S. Singamaneni , “Introduction: Bioinspired and Biomimetic Materials,” Chemical Reviews 117 (2017): 12581–12583, 10.1021/acs.chemrev.7b00552. 29065691

[2] Q. Cheng , L. Jiang , and Z. Tang , “Bioinspired Layered Materials With Superior Mechanical Performance,” Accounts of Chemical Research 47 (2014): 1256–1266, 10.1021/ar400279t. 24635413

[3] S. Palanisamy , M. Kalimuthu , M. Palaniappan , et al., “Characterization of Acacia Caesia Bark Fibers (ACBFs),” Journal of Natural Fibers 19 (2022): 10241–10252, 10.1080/15440478.2021.1993493.

[4] U. G. K. Wegst , H. Bai , E. Saiz , A. P. Tomsia , and R. O. Ritchie , “Bioinspired Structural Materials,” Nature Materials 14 (2015): 23–36, 10.1038/nmat4089. 25344782

[5] X. Peng , B. Zhang , Z. Wang , et al., “Bioinspired Strategies for Excellent Mechanical Properties of Composites,” Journal of Bionic Engineering 19 (2022): 1203–1228, 10.1007/s42235-022-00199-9.

[6] K. Manickaraj , R. Ramamoorthi , R. Karuppasamy , K. R. Sakthivel , and B. Vijayaprakash , “A Review of Natural Biofiber‐Reinforced Polymer Matrix Composites,” in Evolutionary Manufacturing, Design and Operational Practices for Resource and Environmental Sustainability (Scrivener Publishing LLC, 2024): 135–141.

[7] N. Suresh Kumar , R. Padma Suvarna , K. Chandra Babu Naidu , P. Banerjee , A. Ratnamala , and H. Manjunatha , “A Review on Biological and Biomimetic Materials and Their Applications,” Applied Physics A 126 (2020): 445.

[8] X. Yan , B. Bethers , H. Chen , et al., “Recent Advancements in Biomimetic 3D Printing Materials With Enhanced Mechanical Properties,” Frontiers in Materials 8 (2021): 518886, 10.3389/fmats.2021.518886.

[9] B. Mylsamy , K. Aruchamy , S. K. M. Shanmugam , S. Palanisamy , and N. Ayrılmis , “Improving Performance of Composites: Natural and Synthetic Fibre Hybridisation Techniques in Composite Materials—A Review,” Materials Chemistry and Physics 334 (2025): 130439, 10.1016/j.matchemphys.2025.130439.

[10] N. A. Yaraghi and D. Kisailus , “Biomimetic Structural Materials: Inspiration From Design and Assembly,” Annual Review of Physical Chemistry 69 (2018): 23–57, 10.1146/annurev-physchem-040215-112621. 29237136

[11] A. R. Studart , “Towards High‐Performance Bioinspired Composites,” Advanced Materials 24 (2012): 5024–5044, 10.1002/adma.201201471. 22791358

[12] O. Speck and T. Speck , “An Overview of Bioinspired and Biomimetic Self‐Repairing Materials,” Biomimetics 4 (2019): 26, 10.3390/biomimetics4010026. 31105211 PMC6477613

[13] K. Sathish , K. Manickaraj , S. A. Krishna , K. M. Basha , and R. Pravin , “Integrating Sustainable Materials in Exoskeleton Development: A Review,” AIP Conference Proceedings 3221 (2024): 020021, 10.1063/5.0235913.

[14] L. S. Dimas and M. J. Buehler , “Influence of Geometry on Mechanical Properties of Bio‐Inspired Silica‐Based Hierarchical Materials,” Bioinspiration & Biomimetics 7 (2012): 36024, 10.1088/1748-3182/7/3/036024. 22740585

[15] M. Xu , Z. Zhao , P. Wang , S. Duan , H. Lei , and D. Fang , “Mechanical Performance of Bio‐Inspired Hierarchical Honeycomb Metamaterials,” International Journal of Solids and Structures 254‐255 (2022): 111866, 10.1016/j.ijsolstr.2022.111866.

[16] M. S. Ganewatta , Z. Wang , and C. Tang , “Chemical Syntheses of Bioinspired and Biomimetic Polymers Toward Biobased Materials,” Nature Reviews Chemistry 5 (2021): 753–772, 10.1038/s41570-021-00325-x. PMC9555244 36238089

[17] N. San Ha and G. Lu , “A Review of Recent Research on Bio‐Inspired Structures and Materials for Energy Absorption Applications,” Composites Part B: Engineering 181 (2020): 107496.

[18] E. E. de Obaldia , C. Jeong , L. K. Grunenfelder , D. Kisailus , and P. Zavattieri , “Analysis of the Mechanical Response of Biomimetic Materials With Highly Oriented Microstructures Through 3D Printing, Mechanical Testing and Modeling,” Journal of the Mechanical Behavior of Biomedical Materials 48 (2015): 70–85, 10.1016/j.jmbbm.2015.03.026. 25913610

[19] E. Pekhtasheva , E. Mastalygina , I. Leonova , et al., “Investigation of Toxicity in Textile Materials From Natural and Synthetic‐Based Polymers Utilizing Bioassay Performances,” BioResources 20 (2025): 765–789, 10.15376/biores.20.1.765-789.

[20] K. Manickaraj , A. Karthik , S. Palanisamy , et al., “Improving Mechanical Performance of Hybrid Polymer Composites: Incorporating Banana Stem Leaf and Jute Fibers With Tamarind Shell Powder,” BioResources 20 (2025): 1998–2025.

[21] P.‐Y. Chen , J. McKittrick , and M. A. Meyers , “Biological Materials: Functional Adaptations and Bioinspired Designs,” Progress in Materials Science 57 (2012): 1492–1704, 10.1016/j.pmatsci.2012.03.001.

[22] N. San Ha , T. M. Pham , T. T. Tran , H. Hao , and G. Lu , “Mechanical Properties and Energy Absorption of Bio‐Inspired Hierarchical Circular Honeycomb,” Composites Part B: Engineering 236 (2022): 109818.

[23] M. Karuppusamy , R. Ramamoorthi , R. Karuppasamy , and M. Navin , “Review on Fabrication and Applications of Jute Fiber Epoxy Composite Reinforced Bio Composite,” Journal of Advanced Mechanical Sciences 2 3 (2023): 76–81.

[24] E. R. S. Goutham , S. S. Hussain , C. Muthukumar , et al., “Drilling Parameters and Post‐Drilling Residual Tensile Properties of Natural‐Fiber‐Reinforced Composites: A Review,” Journal of Composites Science 7 (2023): 136, 10.3390/jcs7040136.

[25] P. Pandiarajan , P. G. Baskaran , S. Palanisamy , et al., “Enhancing Polyester Composites With Nano Aristida Hystrix Fibers: Mechanical and Microstructural Insights,” BioResources 20 (2025): 9257–9281, 10.15376/biores.20.4.9257-9281.

[26] T. Ramakrishnan , K. Manickaraj , S. P. Prithiv , S. L. Aditya , N. Rajanarayanan , and S. Gopalsamy , “Advancements in Aluminum Metal Matrix Composites: Reinforcement, Manufacturing, and Applications,” AIP Conference Proceedings 3221 (2024): 020030, 10.1063/5.0235881.

[27] X. Zhang , J. Li , C. Ma , H. Zhang , and K. Liu , “Biomimetic Structural Proteins: Modular Assembly and High Mechanical Performance,” Accounts of Chemical Research 56 (2023): 2664–2675, 10.1021/acs.accounts.3c00372. 37738227

[28] E. Munch , M. E. Launey , D. H. Alsem , E. Saiz , A. P. Tomsia , and R. O. Ritchie , “Tough, Bio‐Inspired Hybrid Materials,” Science 322 (2008): 1516–1520, 10.1126/science.1164865. 19056979

[29] G. Ravichandran , K. Ramasamy , K. Manickaraj , et al., “Effect of Sal Wood and Babool Sawdust Fillers on the Mechanical Properties of Snake Grass Fiber‐Reinforced Polyester Composites,” BioResources 20 (2025): 8674–8694, 10.15376/biores.20.4.8674-8694.

[30] K. Sathish , K. Manickaraj , C. Vanchimuthu , V. Thiyagarajan , and C. Bavadharani , “Investigating the Effects of Draft Tube and Its Properties on Francis turbine Performance–A Critical Review,” AIP Conference Proceedings 3221 (2024): 020036, 10.1063/5.0235917.

[31] Y. Liu , J. Ren , and S. Ling , “Bioinspired and Biomimetic Silk Spinning,” Composites Communications 13 (2019): 85–96, 10.1016/j.coco.2019.03.004.

[32] T. Schiller and T. Scheibel , “Bioinspired and Biomimetic Protein‐Based Fibers and Their Applications,” Communications Materials 5 (2024): 56, 10.1038/s43246-024-00488-2.

[33] W. Huang , D. Restrepo , J. Jung , et al., “Multiscale Toughening Mechanisms in Biological Materials and Bioinspired Designs,” Advanced Materials 31 (2019): 1901561, 10.1002/adma.201901561. 31268207

[34] A. J. Shoffstall and J. R. Capadona , “Bioinspired Materials and Systems for Neural Interfacing,” Current Opinion in Biomedical Engineering 6 (2018): 110–119, 10.1016/j.cobme.2018.05.002.

[35] S. Palanisamy , K. Vijayananth , T. M. Murugesan , M. Palaniappan , and C. Santulli , “The Prospects of Natural Fiber Composites: A Brief Review,” International Journal of Lightweight Materials and Manufacture 7 (2024): 496–506, 10.1016/j.ijlmm.2024.01.003.

[36] S. Gokul , T. Ramakrishnan , K. Manickaraj , P. Devadharshan , M. K. Mathew , and T. V. Prabhu , “Analyzing Challenges and Prospects for Sustainable Development With Green Energy: A Comprehensive Review,” AIP Conference Proceedings 3221 (2024): 020043, 10.1063/5.0235884.

[37] Z. Xia , Biomimetic Principles and Design of Advanced Engineering Materials (John Wiley & Sons, 2016), 10.1002/9781118926253.

[38] S. Palanisamy , T. M. Murugesan , M. Palaniappan , C. Santulli , N. Ayrilmis , and A. Alavudeen , “Selection and Processing of Natural Fibers and Nanocellulose for Biocomposite Applications: A Brief Review,” BioResources 19 (2024): 1789.

[39] O. Z. Fisher , A. Khademhosseini , R. Langer , and N. A. Peppas , “Bioinspired Materials for Controlling Stem Cell Fate,” Accounts of Chemical Research 43 (2010): 419–428, 10.1021/ar900226q. 20043634 PMC2840210

[40] F. Barthelat , J. E. Rim , and H. D. Espinosa , “A Review on the Structure and Mechanical Properties of Mollusk Shells–Perspectives on Synthetic Biomimetic Materials,” in Applied Scanning Probe Methods XIII: Biomimetics and Industrial Applications (Springer, 2009), 17–44, 10.1007/978-3-540-85049-6.

[41] K. Manickaraj , T. Nithyanandhan , K. Sathish , R. Karuppasamy , and B. Sachuthananthan , “An Experimental Investigation of Volume Fraction of Natural Java Jute and Sponge Gourd Fiber Reinforced Polymer Matrix Composite,” in 2024 10th International Conference on Advanced Computing and Communication Systems (ICACCS) (IEEE, 2024), 2373–2378.

[42] Z.‐L. Yu , N. Yang , L.‐C. Zhou , et al., “Bioinspired Polymeric woods,” Science advances 4 (2018): aat7223. 10.1126/sciadv.aat7223 PMC6086613 30105307

[43] M. Gurusamy , S. Soundararajan , M. Karuppusamy , and K. Ramasamy , “Exploring the Mechanical Impact of Fine Powder Integration From Ironwood Sawdust and COCO Dust Particles in Epoxy Composites,” Matéria (Rio de Janeiro) 29 (2024): 20240216.

[44] J. Ren , Y. Wang , Y. Yao , et al., “Biological Material Interfaces as Inspiration for Mechanical and Optical Material Designs,” Chemical Reviews 119 (2019): 12279–12336, 10.1021/acs.chemrev.9b00416. 31793285

[45] I. Tunn , M. J. Harrington , and K. G. Blank , “Bioinspired Histidine–Zn^2+^ Coordination for Tuning the Mechanical Properties of Self‐Healing Coiled Coil Cross‐Linked Hydrogels,” Biomimetics 4 (2019): 25, 10.3390/biomimetics4010025. 31105210 PMC6477626

[46] K. Aruchamy , M. Karuppusamy , S. Krishnakumar , et al., “Enhancement of Mechanical Properties of Hybrid Polymer Composites Using Palmyra Palm and Coconut Sheath Fibers: The Role of Tamarind Shell Powder,” BioResources 20 (2025): 698–724, 10.15376/biores.20.1.698-724.

[47] B. Natarajan and J. W. Gilman , “Bioinspired Bouligand Cellulose Nanocrystal Composites: A Review of Mechanical Properties,” Philosophical Transactions of the Royal Society A: Mathematical, Physical and Engineering Sciences 376 (2018): 20170050. 10.1098/rsta.2017.0050 PMC5746561 29277746

[48] S. Palanisamy , M. Kalimuthu , R. Nagarajan , J. M. Fernandes Marlet , and C. Santulli , “Physical, Chemical, and Mechanical Characterization of Natural Bark Fibers (NBFs) Reinforced Polymer Composites: A Bibliographic Review,” Fibers 11 (2023): 13.

[49] S. Iravani and R. S. Varma , “Bioinspired and Biomimetic MXene‐Based Structures With Fascinating Properties: Recent Advances,” Materials Advances 3 (2022): 4783–4796, 10.1039/D2MA00151A.

[50] N. Thangavel , N. K. Shanmugavel , M. Karuppusamy , and R. Thirumalaisamy , “Friction and Wear Behavior of Premixed Reinforcement Hybrid Composite Materials,” Matéria (Rio de Janeiro) 29 (2024): 20240552.

[51] M. A. A. Abdelhamid and S. P. Pack , “Biomimetic and Bioinspired Silicifications: Recent Advances for Biomaterial Design and Applications,” Acta Biomaterialia 120 (2021): 38–56, 10.1016/j.actbio.2020.05.017. 32447061

[52] T. Nithyanandhan , K. Manickaraj , K. Sathish , N. Ramachandran , and B. Sachuthananthan , “Effects of Palm Stalk Ash on Mechanical Properties of Al6061 Reinforced With Graphite by Using Stir Casting Process,” in 2024 10th International Conference on Advanced Computing and Communication Systems (ICACCS) (IEEE, 2024), 2357–2364.

[53] N. H. Alrasheedi , P. Sivasubramanian , M. Karuppusamy , B. Haldar , and T. K. Durairaj , “Hybrid Bio‐Composites Reinforced With Kenaf and Snake Grass Fibers and Neem Gum: Synergistic Effects and Role of Fiber Aspect Ratio,” BioResources 21 (2026): 459–481, 10.15376/biores.21.1.459-481.

[54] M. C. Demirel , M. Cetinkaya , A. Pena‐Francesch , and H. Jung , “Recent Advances in Nanoscale Bioinspired Materials,” Macromolecular bioscience 15 (2015): 300–311. 25476469 10.1002/mabi.201400324

[55] B. Mylsamy , S. K. M. Shanmugam , K. Aruchamy , S. Palanisamy , R. Nagarajan , and N. Ayrilmis , “A Review on Natural Fiber Composites: Polymer Matrices, Fiber Surface Treatments, Fabrication Methods, Properties, and Applications,” Polymer Engineering & Science 64 (2024): 2345–2373, 10.1002/pen.26713.

[56] P. Rajamani , V. Selvaraj , M. Velusamy , M. Karuppusamy , R. Thirumalaisamy , and K. Radhakrishnan , “Effect of Packing Factor on Energy Performance of Solar PV/T Water‐Heating Collector System–An Experimental Study,” AIP Conference Proceedings 3231 (2024): 020007, 10.1063/5.0235849.

[57] J. D. Kechagias , K. Ninikas , F. Vakouftsi , N. A. Fountas , S. Palanisamy , and N. M. Vaxevanidis , “Optimization of Laser Beam Parameters During Processing of ASA 3D‐Printed Plates,” The International Journal of Advanced Manufacturing Technology 130 (2023): 527–539.

[58] M. A. Meyers and P.‐Y. Chen , Biological Materials Science: Biological Materials, Bioinspired Materials and Biomaterials (Cambridge University Press, 2015).

[59] U. Shashikumar , P.‐C. Tsai , C.‐T. Wang , C.‐H. Lay , and V. K. Ponnusamy , “Beyond Biomimicry: Innovative Bioinspired Materials Strategies and Perspectives for High‐Performance Energy Storage Devices,” Process Safety and Environmental Protection 191 (2024): 1193–1217, 10.1016/j.psep.2024.08.123.

[60] L.‐F. Chen , S.‐X. Ma , S. Lu , et al., “Biotemplated Synthesis of Three‐Dimensional Porous MnO/C‐N Nanocomposites From Renewable Rapeseed Pollen: An Anode Material for Lithium‐Ion Batteries,” Nano Research 10 (2017): 1–11, 10.1007/s12274-016-1283-7.

[61] S. Zhao and J.‐H. Ahn , “Rational Design of High‐Performance Wearable Tactile Sensors Utilizing Bioinspired Structures/Functions, Natural Biopolymers, and Biomimetic Strategies,” Materials Science and Engineering: R: Reports 148 (2022): 100672, 10.1016/j.mser.2022.100672.

[62] Y. Zhou , K. Liu , and H. Zhang , “Biomimetic Mineralization: From Microscopic to Macroscopic Materials and Their Biomedical Applications,” ACS Applied Bio Materials 6 (2023): 3516–3531, 10.1021/acsabm.3c00109. 36944024

[63] F. Barthelat , “Biomimetics for Next Generation Materials,” Philosophical Transactions of the Royal Society A: Mathematical, Physical and Engineering Sciences 365 (2007): 2907–2919, 10.1098/rsta.2007.0006. 17855221

[64] Z. Chen , Z. Wang , and Z. Gu , “Bioinspired and Biomimetic Nanomedicines,” Accounts of Chemical Research 52 (2019): 1255–1264, 10.1021/acs.accounts.9b00079. 30977635 PMC7293770

[65] T. Nithyanandhan , P. Sivaraman , K. Manickaraj , N. M. Raj , M. S. Pragash , and A. Tharun , “Tribological Behaviour of Aluminium 6061 Reinforced With Graphite and Chicken Bone Ash by Using Stir Casting,” International Journal of Vehicle Structures and Systems 14 (2022): 849–854, 10.4273/ijvss.14.7.04.

[66] G. Karuppiah , K. C. Kuttalam , N. Ayrilmis , et al., “Tribological Analysis of Jute/Coir Polyester Composites Filled With Eggshell Powder (ESP) or Nanoclay (NC) Using Grey Rational Method,” Fibers 10 (2022): 60.

[67] Y. Zhang , H. Yao , C. Ortiz , J. Xu , and M. Dao , “Bio‐Inspired Interfacial Strengthening Strategy Through Geometrically Interlocking Designs,” Journal of the Mechanical Behavior of Biomedical Materials 15 (2012): 70–77, 10.1016/j.jmbbm.2012.07.006. 23032427

[68] H. Zhao , W. Dong , Y. Deng , et al., “Biomass‐Based Biomimetic‐Oriented Janus Nanoarchitecture for Efficient Heavy‐Metal Enrichment and Interfacial Solar Water Sanitation,” Interdisciplinary Materials 1 (2022): 537–547, 10.1002/idm2.12057.

[69] L. Ren , X. Zhou , Q. Liu , et al., “3D Magnetic Printing of Bio‐Inspired Composites With Tunable Mechanical Properties,” Journal of Materials Science 53 (2018): 14274–14286, 10.1007/s10853-018-2447-5.

[70] P. R. Govindarajan , R. Shanmugavel , S. Palanisamy , et al., “Crashworthiness Analysis and Morphology of Hybrid Hollow Tubes Reinforced by Aluminum Mesh With Hybrid Woven Fibre Composites (Basalt, Jute, Hemp, Banana, Bamboo) Using Roll‐Wrapping Technique,” BioResources 19 (2024): 6584.

[71] N. Ayrılmıs , E. Yurttas , N. Tetik , et al., “Antibacterial Performance of Biodegradable Polymer and Hazelnut Husk Flour Antibacterial Biofilm With Silver Nanoparticles,” BioResources 19 (2024): 8812–8826.

[72] K. Manickaraj , R. Ramamoorthi , S. Sathish , and M. Makeshkumar , “Effect of Hybridization of Novel African Teff and Snake Grass Fibers Reinforced Epoxy Composites With Bio Castor Seed Shell Filler: Experimental Investigation,” Polymers & Polymer Composites 30 (2022): 1–11.

[73] Z. Zhang , Z. Mu , Y. Wang , et al., “Lightweight Structural Biomaterials With Excellent Mechanical Performance: A Review,” Biomimetics 8 (2023): 153, 10.3390/biomimetics8020153. 37092405 PMC10123704

[74] S. Cui , Z. Lu , and Z. Yang , “Effect of Interlocking Structure on Mechanical Properties of Bio‐Inspired Nacreous Composites,” Composite Structures 226 (2019): 111260, 10.1016/j.compstruct.2019.111260.

[75] W. Li , Y. Pei , C. Zhang , and A. G. P. Kottapalli , “Bioinspired Designs and Biomimetic Applications of Triboelectric Nanogenerators,” Nano Energy 84 (2021): 105865, 10.1016/j.nanoen.2021.105865.

[76] S. Kilper , S. J. Facey , Z. Burghard , B. Hauer , D. Rothenstein , and J. Bill , “Macroscopic Properties of Biomimetic Ceramics Are Governed by the Molecular Recognition at the Bioorganic–Inorganic Interface,” Advanced Functional Materials 28 (2018): 1705842, 10.1002/adfm.201705842.

[77] P. Sivasubramanian , M. Kalimuthu , M. Palaniappan , A. Alavudeen , N. Rajini , and C. Santulli , “Effect of Alkali Treatment on the Properties of Acacia Caesia Bark Fibres,” Fibers 9 (2021): 49.

[78] V. P. Thompson , “The Tooth: An Analogue for Biomimetic Materials Design and Processing,” Dental Materials 36 (2020): 25–42, 10.1016/j.dental.2019.08.106. 31543376

[79] P. Makvandi , A. Maleki , M. Shabani , et al., “Bioinspired Microneedle Patches: Biomimetic Designs, Fabrication, and Biomedical Applications,” Matter 5 (2022): 390–429.

[80] T. A. Kurniawan , X. Liang , H. H. Goh , et al., “Leveraging Food Waste for Electricity: A Low‐Carbon Approach in Energy Sector for Mitigating Climate Change and Achieving Net Zero Emission in Hong Kong (China),” Journal of Environmental Management 351 (2024): 1–13, 10.1016/j.jenvman.2023.119879. 38157574

[81] J. Sun and B. Bhushan , “Structure and Mechanical Properties of Beetle Wings: A Review,” RSC Advances 2 (2012): 12606–12623, 10.1039/c2ra21276e.

[82] L. Montero de Espinosa , W. Meesorn , D. Moatsou , and C. Weder , “Bioinspired Polymer Systems With Stimuli‐Responsive Mechanical Properties,” Chemical Reviews 117 (2017): 12851–12892, 10.1021/acs.chemrev.7b00168. 28752995

[83] C. Zhang , D. A. Mcadams , and J. C. Grunlan , “Nano/Micro‐Manufacturing of Bioinspired Materials: A Review of Methods to Mimic Natural Structures,” Advanced Materials 28 (2016): 6292–6321, 10.1002/adma.201505555. 27144950

[84] K. Manickaraj , R. Ramamoorthi , R. Karuppasamy , S. Kannan , and B. Vijayaprakash , “Experimental Investigation of Steel and Porous Al Foam LM Vehicle Leaf Spring by Using Mechanical and Computer Method,” Evolutionary Manufacturing, Design and Operational Practices for Resource and Environmental Sustainability (Scrivener Publishing LLC, 2024): 107–112.

[85] B. Wang , W. Yang , J. McKittrick , and M. A. Meyers , “Keratin: Structure, Mechanical Properties, Occurrence in Biological Organisms, and Efforts at Bioinspiration,” Progress in Materials Science 76 (2016): 229–318, 10.1016/j.pmatsci.2015.06.001.

[86] R. G. Padmanabhan , S. Rajesh , S. Karthikeyan , et al., “Evaluation of Mechanical Properties and Fick's Diffusion Behaviour of Aluminum‐DMEM Reinforced With Hemp/Bamboo/Basalt Woven Fiber Metal Laminates (WFML) Under Different Stacking Sequences,” Ain Shams Engineering Journal 15 (2024): 102759, 10.1016/j.asej.2024.102759.

[87] B. Wang , J. G. Torres‐Rendon , J. Yu , Y. Zhang , and A. Walther , “Aligned Bioinspired Cellulose Nanocrystal‐Based Nanocomposites With Synergetic Mechanical Properties and Improved Hygromechanical Performance,” ACS Applied Materials & Interfaces 7 (2015): 4595–4607, 10.1021/am507726t. 25646801

[88] M. Sarikaya , H. Fong , N. Sunderland , et al., “Biomimetic Model of a Sponge‐Spicular Optical Fiber—Mechanical Properties and Structure,” Journal of Materials Research 16 (2001): 1420–1428, 10.1557/JMR.2001.0198.

[89] S. K. Palaniappan , K. Aruchamy , M. Bhuvaneshwaran , T. Velayutham , and K. Manickaraj , “Polyacrylonitrile Fiber: Composites and Applications,” in Synthetic and Mineral Fibers, Their Composites and Applications (Elsevier, 2024), 269–290.

[90] O. Speck and T. Speck , “Functional Morphology of Plants—A Key to Biomimetic Applications,” New Phytologist 231 (2021): 950–956, 10.1111/nph.17396. 33864693

[91] N. V. Chithra , R. Karuppasamy , K. Manickaraj , and T. Ramakrishnan , “Effect of Reinforcement Addition on Mechanical Behavior of Al MMC‐A Critical Review,” Journal of Environmental Nanotechnology 13 (2024): 65–79, 10.13074/jent.2024.06.242632.

[92] A. Svagan , M. A. S. Azizi Samir , and L. A. Berglund , “Biomimetic Foams of High Mechanical Performance Based on Nanostructured Cell Walls Reinforced by Native Cellulose Nanofibrils,” Advanced Materials 20 (2008): 1263–1269, 10.1002/adma.200701215.

[93] S. Palanisamy , M. Kalimuthu , N. Rajini , and C. Santulli , “Biocomposites Derived From Plant Fiber Resources,” in Biocomposites – Bio‐Based Fibres and Polymers From Renewable Resources, (Woodhead Publishing, 2024): 23–54.

[94] O. Paris , I. Burgert , and P. Fratzl , “Biomimetics and Biotemplating of Natural Materials,” MRS Bulletin 35 (2010): 219–225, 10.1557/mrs2010.655.

[95] M. Peng , Z. Wen , L. Xie , et al., “3D Printing of Ultralight Biomimetic Hierarchical Graphene Materials With Exceptional Stiffness and Resilience,” Advanced Materials 31 (2019): 1902930, 10.1002/adma.201902930. 31267581

[96] K. Manickaraj , R. Ramamoorthi , T. Ramakrishnan , and R. Karuppasamy , “Enhancing Solid Waste Sustainability With Iroko Wooden Sawdust and African Oil Bean Shell Particle‐Strengthened Epoxy Composites,” Global NEST Journal 25 (2024): 1–5.

[97] M. Gagliardi , “Biomimetic and Bioinspired Nanoparticles for Targeted Drug Delivery,” Therapeutic Delivery 8 (2017): 289–299, 10.4155/tde-2017-0013. 28361608

[98] A. Karthik , M. Bhuvaneshwaran , M. S. Senthil Kumar , S. Palanisamy , M. Palaniappan , and N. Ayrilmis , “A Review on Surface Modification of Plant Fibers for Enhancing Properties of Biocomposites,” ChemistrySelect 9 (2024): 202400650, 10.1002/slct.202400650.

[99] P. Egan , R. Sinko , P. R. LeDuc , and S. Keten , “The Role of Mechanics in Biological and Bio‐Inspired Systems,” Nature Communications 6 (2015): 7418, 10.1038/ncomms8418. 26145480

[100] G. X. Gu , C.‐T. Chen , D. J. Richmond , and M. J. Buehler , “Bioinspired Hierarchical Composite Design Using Machine Learning: Simulation, Additive Manufacturing, and Experiment,” Materials Horizons 5 (2018): 939–945, 10.1039/C8MH00653A.

[101] R. Xiong , W. Wu , C. Lu , and H. Cölfen , “Bioinspired Chiral Template Guided Mineralization for Biophotonic Structural Materials,” Advanced Materials 34 (2022): 2206509, 10.1002/adma.202206509. 36208076

[102] P. A. Guerette , S. Hoon , Y. Seow , et al., “Accelerating the Design of Biomimetic Materials by Integrating RNA‐Seq With Proteomics and Materials Science,” Nature Biotechnology 31 (2013): 908–915, 10.1038/nbt.2671. 24013196

[103] Y. Chen , B. Dang , C. Wang , et al., “Intelligent Designs From Nature: Biomimetic Applications in Wood Technology,” Progress in Materials Science 139 (2023): 101164, 10.1016/j.pmatsci.2023.101164.

[104] Y. Alex , N. Divakaran , and P. V. A. Kumar , “Computational Studies and Modeling Aspects of Functionalized Polymer nanocomposites,” Advances in Functionalized Polymer Nanocomposites (Woodhead Publishing, 2024), 1001–1030.

[105] J. Ubaid , B. L. Wardle , and S. Kumar , “Bioinspired Compliance Grading Motif of Mortar in Nacreous Materials,” ACS Applied Materials & Interfaces 12 (2020): 33256–33266, 10.1021/acsami.0c08181. 32559363

[106] P. Hajibabaee , F. Pourkamali‐Anaraki , and M. A. Hariri‐Ardebili , “Adaptive Conformal Prediction Intervals Using Data‐Dependent Weights with Application to Seismic Response Prediction,” IEEE Access 12 (2024): 53579–53597, 10.1109/ACCESS.2024.3387858.

